# *Echinacea purpurea* ameliorates Bleomycin-induced pulmonary fibrosis in rats through modulating NADPH oxidase-4 and endothelin-1/connective tissue growth factor/matrix metalloproteinases signalling axis

**DOI:** 10.1038/s41598-025-06559-9

**Published:** 2025-09-02

**Authors:** Abeer A. A. Salama, Rania Elgohary, Sahar Abd Elwahab, Rasha E. Mostafa

**Affiliations:** 1 Pharmacology Department, Medical Research and Clinical Studies Institute, National Research Centre (ID: 60014618), 33 ELBohouth St. (former EL Tahrir St.), P.O. 12622, Dokki, Cairo Egypt; 2 Narcotics, Ergogenics and Poisons Department, Medical Research and Clinical Studies Institute, National Research Centre, Dokki, Cairo Egypt; 3 Medical Biochemistry and Molecular Biology Department, Faculty of Medicine, Cairo University, Cairo, Egypt

**Keywords:** Echinacea, Bleomycin, Lung fibrosis, NOX4, Endothelin-1, CTGF, Biochemistry, Molecular biology, Cardiology, Diseases, Medical research

## Abstract

Idiopathic pulmonary fibrosis (IPF) is one of the rapidly progressing interstitial lung illnesses. Bleomycin (Bleo) is used as a chemotherapeutic agent for the treatment of lymphoma patients. The major side effects of Bleo include lung fibrosis, characterized by the accumulation of inflammatory cells. *Echinacea purpurea* (ECH) possesses immune-modulating, antiviral, antimicrobial and anti-inflammatory activities. The current study aims to evaluate the possible protective effects of ECH against Bleomycin-induced pulmonary fibrosis. Forty rats were divided into four groups (*n* = 10). Group I represented the normal-control group. Group II represented the Bleo-control group. Groups III and IV received intra-tracheal Bleo followed by oral ECH (25 and 50 mg/kg); respectively, for 1 month. Lung tissue contents of reduced glutathione (GSH), malondialdehyde (MDA), transforming growth factor-beta (TGF-β), matrix metalloproteinases (MMP-2 & MMP-9), tissue inhibitor of metalloproteinase 1 (TIMP-1), MMP-9/TIMP-1 ratio, collagen-1 and alpha-Smooth muscle actin (α-SMA) were measured. NADPH oxidase 4 (NOX4), connective tissue growth factor (CTGF) and endothelin-1 (ET-1) genes were quantified using PCR. Moreover, lung tissue histopathological changes were screened. Intra-tracheal Bleo instillation resulted in significant increments in the lung tissue contents of MDA, TGF-β, MMP-2 & MMP-9, TIMP-1, MMP-9/TIMP-1 ratio, collagen-1 and α-SMA. Moreover, Bleo significantly elevated the PCR expression of NOX4, CTGF and ET-1 genes in lung tissues and caused apparent lung tissue histopathological fibrotic changes. ECH treatment ameliorated all the aforementioned parameters and mitigated the lung tissue histopathological fibrotic changes induced by Bleo. The study highlighted for the first time the anti-oxidant, anti-inflammatory and anti-fibrotic effects of ECH against Bleo-induced pulmonary fibrosis in rats. The study suggests that these effects are mainly mediated via the modulation of Gelatinases, NOX4, ET-1 and CTGF. Accordingly, ECH is anticipated as a potential therapy to be added to the treatment regimen of pulmonary fibrosis.

## Introduction

Lung diseases are major worldwide causes of illness and death. Asthma, chronic obstructive pulmonary disease (COPD), pneumonia, idiopathic pulmonary fibrosis (IPF) and lung cancer are amongst the most frequent lung illnesses^1^. IPF, previously described as cryptogenic fibrosing alveolitis, typically affects adults over 40 and is characterized by persistent inflammation of the pulmonary parenchyma^2^. Persistent alveolar inflammation leads to pulmonary toxicity, fibroblast activation, and subsequently fibrosis^3^. Every year, this lethal illness results in more than one million fatalities in the US and more than five million worldwide. To date, the root cause of IPF is not well defined. Few effective treatments for IPF are currently available^4^. Antiinflammatories, immunosuppressives and anti-inflammatory interleukins have largely failed to treat pulmonary fibrosis. Treatment approaches have changed to focus on anti-fibrotic agents as the pathogenic actions of fibrotic foci, along with fibroblast proliferation have come to light. The use of these drugs was mostly justified by data pointing to abnormalities in the extracellular matrix deposition, increased fibroblast proliferation, myofibroblast activation, and alveolar inflammatory cytokine imbalance in the lungs of patients suffering IPF^5^.

Recent investigations report that inflammatory cells can secrete specific growth factors, including epidermal growth factor, platelet-derived growth factor, along with tissue growth factor-beta1 (TGF-β1). Alterations of intestinal microbiota may lead to inflammation^6^. Reactive oxygen species (ROS), pro-inflammatory cytokines, proteases, and peroxidases are also released, leading to lung tissue damage. Inflammation and lung injury are mostly caused by ROS, which is released by active leukocytes^7^. TGF-1β is the major player in IPF^8^. In the lungs, TGF-1β activates inflammatory cells, causing significant ROS production and release as well as damage to epithelial cells. Fibroblasts and myofibroblasts are formed from damaged epithelial cells. These fibroblasts and myofibroblasts build up in the alveolar wall where they deposit, multiply, and regenerate the extracellular matrix by producing extracellular collagen and alpha-smooth muscle actin. This leads to a positive loop causing the progression of fibrosis^9^.

Other causes of IPF include bacterial and viral infections, including the herpes virus, TB, rheumatoid arthritis, radiotherapy for cancer treatment, some mineral substances like asbestos and silica, along with side effects of some chemotherapeutic agents like Bleomycin (Bleo)^10^.

Endothelin-1 (ET-1) is a potent vasoactive peptide that plays a multifaceted role in the pathogenesis of lung fibrosis via promoting fibroblast activation and inflammation. Its involvement in these processes makes it a key player in the development and progression of pulmonary fibrosis. Targeting ET-1 signaling may offer a promising therapeutic strategy for treating fibrosis, particularly when combined with other anti-fibrotic agents that address different aspects of the disease^11^.

The NADPH oxidases (NOXs) family has been linked to pulmonary fibrosis^12^. Recent data suggest that matrix metalloproteinases (MMPs), NOX4 and connective tissue growth factor (CTGF) are upregulated in many fibrotic disorders, including pulmonary fibrosis^13^.

Bleomycin is an antimicrobial drug used as a chemotherapeutic agent for the treatment of many cancers including lymphomas, cervical, vaginal, skin, rectal and pyloric cancers. The use of Bleo leads to increased oxidative stress mainly via reducing the antioxidant enzyme activities. The major side effects of Bleo include lung fibrosis characterized by the accumulation of inflammatory cells (lymphocytes, neutrophils, eosinophils and macrophages) in the bronchoalveolar lavage^14^. Bleo is widely used in animal models for the induction of lung inflammation and fibrosis^3^.

Echinacea (ECH), *Echinacea purpurea*, family Asteraceae, is a perennial blooming herb mainly present in many regions of the US, Canada, and Europe. It is cultivated for both its aesthetic appeal and its well-documented therapeutic virtues. ECH’s chemical makeup plays a variety of roles in influencing its action. The most important chemical constituents include high molecular weight polysaccharides, volatile terpenes, phenolic chemicals, glycoproteins, and derivatives of caffeic acid. ECH has wound-healing, immune-modulating, antiviral, anti-microbial and anti-inflammatory activities. Respiratory issues brought on by bacterial infections can be safely treated with it^15^. ECH has recently been proposed as a potential herbal medicinal option for new coronaviruses, among many other herbal remedies^16^.

Thus, the current work aims to evaluate the possible anti-oxidant and anti-fibrotic effects of ECH against Bleomycin-induced pulmonary fibrosis in rats. The molecular mechanisms underlying these effects will be evaluated, shedding light on the involvement of NOX-4 and ET-1/CTGF/MMPs signalling.

## Materials and methods

### Animals

Male albino Wistar rats (200–300 g) were procured from the animal house of the National Research Centre (Cairo, Egypt) and allocated in plastic cages under controlled environmental conditions (24 ± 2 °C & normal light/dark cycle) with food and water ad libitum. Rats were adapted to the experimental conditions for 7 days before starting the experiment.

### Ethical statement

All experimental procedures adhere to the ARRIVE guidelines and were performed following the recommendations of the National Institutes of Health Guide for Care and Use of Laboratory Animals (NIH Publications No. 8023, revised 1978) and performed as per the ethical standards laid down in the 1964 Declaration of Helsinki and its later amendments. Ethical approval was obtained from the Institutional Animal Ethics Committee (Medical Research Ethics Committee (MREC) of the NRC, Cairo, Egypt; Ethical Approval Number: 5411022023.

### Chemicals and kits

Bleomycin vials (Bleocip 15^®^) were purchased from Cipla Pharmaceutical Company (Mumbai, India). *Echinacea purpurea* (Immulant^®^, 175 mg capsules) was obtained from MEPACO-MEDIFOOD (Cairo, Egypt).

Transforming growth factor-beta (TGF-β; Catalog number: SL0709Ra), Matrix metalloproteinases 2 (MMP-2; Catalog number: SL0483Ra), Matrix metalloproteinases 9 (MMP-9; Catalog number: SL0490Ra), Tissue Inhibitor of metalloproteinase 1 (TIMP-1; Catalog number: SL0692Ra), Collagen-1 (Catalog number: SL0181Ra) and alpha-Smooth muscle actin (α-SMA; Catalog number: SL0988Ra) were purchased from Sunlong Biotech Co., Ltd, China. All other chemicals were of the highest available analytical grade.

### Induction of pulmonary fibrosis

Thiopental sodium was used to anesthetize rats (50 mg/kg, i.p.), then pulmonary fibrosis was induced by a single Bleo dose (5 mg/kg; intra-tracheal instillation). The trachea was exposed horizontally after a midline neck incision, and the position of the needle was modified until it completely entered the trachea before Bleo was injected. To ensure that Bleo was distributed uniformly throughout the lung tissues, rats were maintained in a vertical position and rotated numerous times^3^.

### Experimental design

**Fig. 1:**
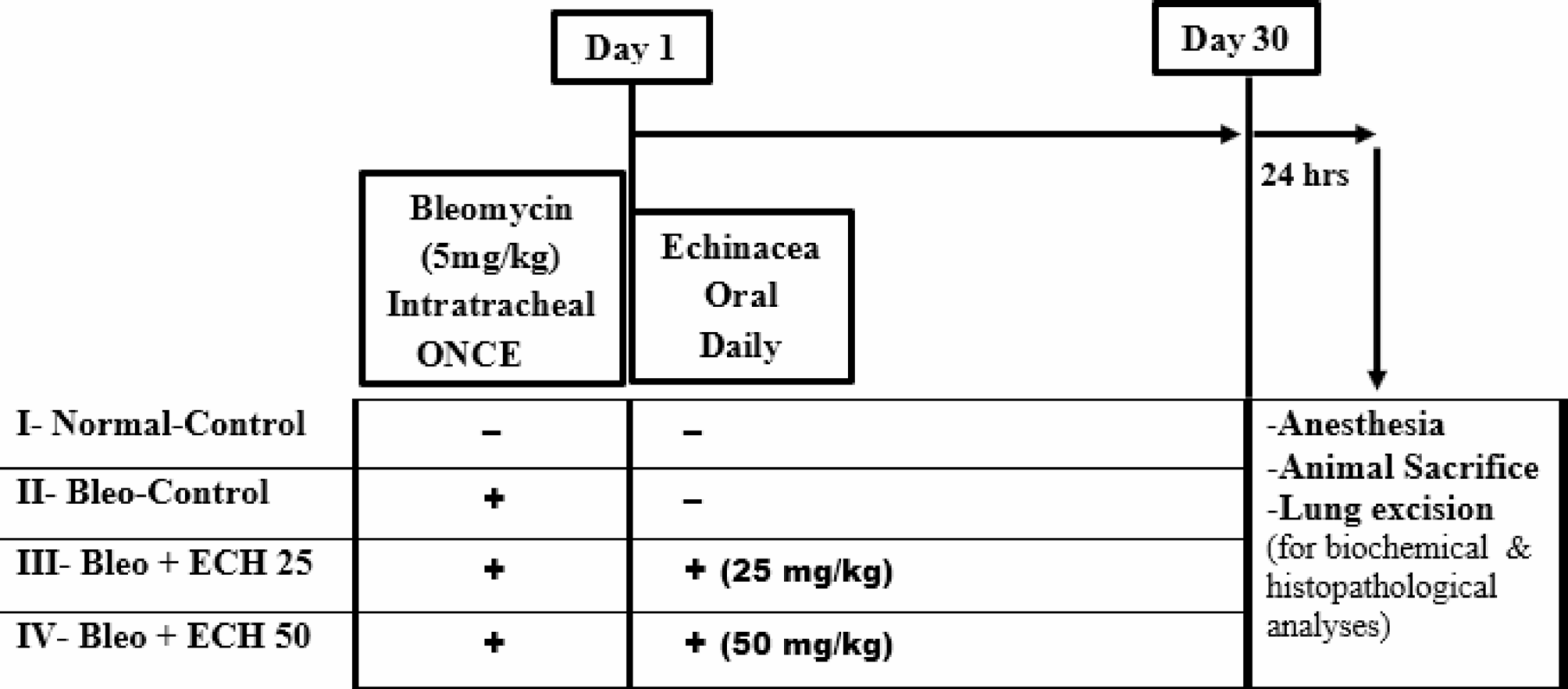
Schematic diagram of experimental design and treatment protocol.

Forty rats were randomly allocated into four groups (*n* = 10). Group I received only saline (5 mL/kg; intra-tracheal), was sham-operated and represents the normal-control group. Pulmonary fibrosis was induced in the remaining groups using Bleo (5 mg/kg). Group II represents the Bleo-control group that received only Bleo. Groups III and IV received intra-tracheal Bleo followed by oral ECH (25 and 50 mg/kg); respectively, starting from day 1 for 1 month^17^. Figure 1 illustrates a schematic flowchart of the study experimental design.

### Biochemical analysis

At the end of the experiment, rats were sacrificed by decapitation under Thiopental sodium anesthesia (50 mg/kg, i.p). The lungs were excised and 0.3 mg of each right lung was homogenized in 5% normal saline to obtain a 20% homogenate. The homogenate was centrifuged for 10 min using a cooling centrifuge at -4^°^ c at 3000 r.p.m, and the supernatant was used for the biochemical analyses. The left lung was used for the histopathological examination.

The concentrations of lipid peroxidation products Malondialdehyde (MDA) and reduced glutathione (GSH) were detected in lung homogenates spectrophotometrically at wavelengths 535 nm and 412 nm according to the methods by Nair and Turner (1984) and Ellman (1959)^18,19^ and; respectively, as described before^20^.

### Quantitative real-time PCR gene expression of NOX4, CTGF and ET-1 in lung tissues

Total RNA was extracted from rats` lung tissue homogenates using TRI reagent (Molecular Research Center Inc., Cincinnati, OH)^21^. Quantification of the extracted RNA was done by spectro-photometry (JENWAY, USA) at 260 nm. The obtained RNA is utilized for the assessment of NADPH oxidase 4 (NOX4), connective tissue growth factor (CTGF) and endothelin-1 (ET-1) gene expression levels with quantitative RT-PCR in agreement with the manufacturer`s protocol (GoTaq^®^ 1-Step RT-qPCR System). PCR primers were designed with Gene Runner Software (Hastings Software Inc., Hastings, NY, USA) with RNA sequences obtained from GenBank, using GAPDH as a housekeeping gene (Table 1). The obtained data were calculated using the 2 − ΔΔ CT method^22^.

**Table 1:** Primer sequence of housekeeping and target genes.

Gene	Forward primer	Reverse primer
NOX4	5′-AGTCAAACAGATGGGA-3′	5′-TGTCCCATATGAGTTGTT-3′
CTGF	5′-TGTGAAGACATACAGGGCTAA-3′	5′-GTTCTCACTTTGGTGGGATAG-3′
ET-1	5′-GAACATCTGTCCGGCTTCTAC-3′	5′-TATGGAATCTCCTGGCTCTCT-3′
GAPDH	5′-TGAACGGGAAGCTCACTGG-3′	5′-TCCACCACCCTGTTGCTGTA-3′

### Histopathological examination

Lung specimens from all animals were dissected immediately after death and fixed in a 10% neutral-buffered formalin solution for at least 12 h. All the specimens were washed and then dehydrated in ascending grades of alcohol, cleared in xylene and embedded in paraffin. Serial Sections 3 μm thick were cut and stained with Haematoxylin and eosin for histopathological examination^23^. Images were captured using an image analysis system with Olympus CX41 light microscope and SC100 video camera attached to a computer system. Photomicrographs were taken at different magnifications and processed using Adobe Photoshop version8.0.

Histopathological scoring of the H&E tissue was done. Four histopathological categories were selected: (1) dilatation of the respiratory tract, (2) infiltration of inflammatory cells, (3) proliferation of respiratory epithelium, and (4) vascular congestion. The first three categories were assessed using a semi-quantitative scale (0 to 3), with 0 indicating no pathology and 3 indicating the most serious damage; the last category was graded from 0 to 1 based on the lack or presence of vascular congestion. The overall histopathological score of the lung was computed by adding the values given for each criterion^24^.

### Statistical analysis

The results were presented as mean ± standard error of means. Shapiro-Wilk test was used for testing normality. A one-way analysis of variance (ANOVA), followed by *Tukey’s* post hoc test, was used to test the statistical significance. Bartlett’s test and Brown-Forsythe test were used for testing the homogeneity of variances. Statistics and graphical presentations were created using GraphPad prism^®^ software (version 9.00 for Windows, San Diego, CA, USA).

## Results

### *Echinacea ameliorates* the oxidative stress parameters in Bleomycin-induced pulmonary fibrosis in rats

**Fig. 2:**
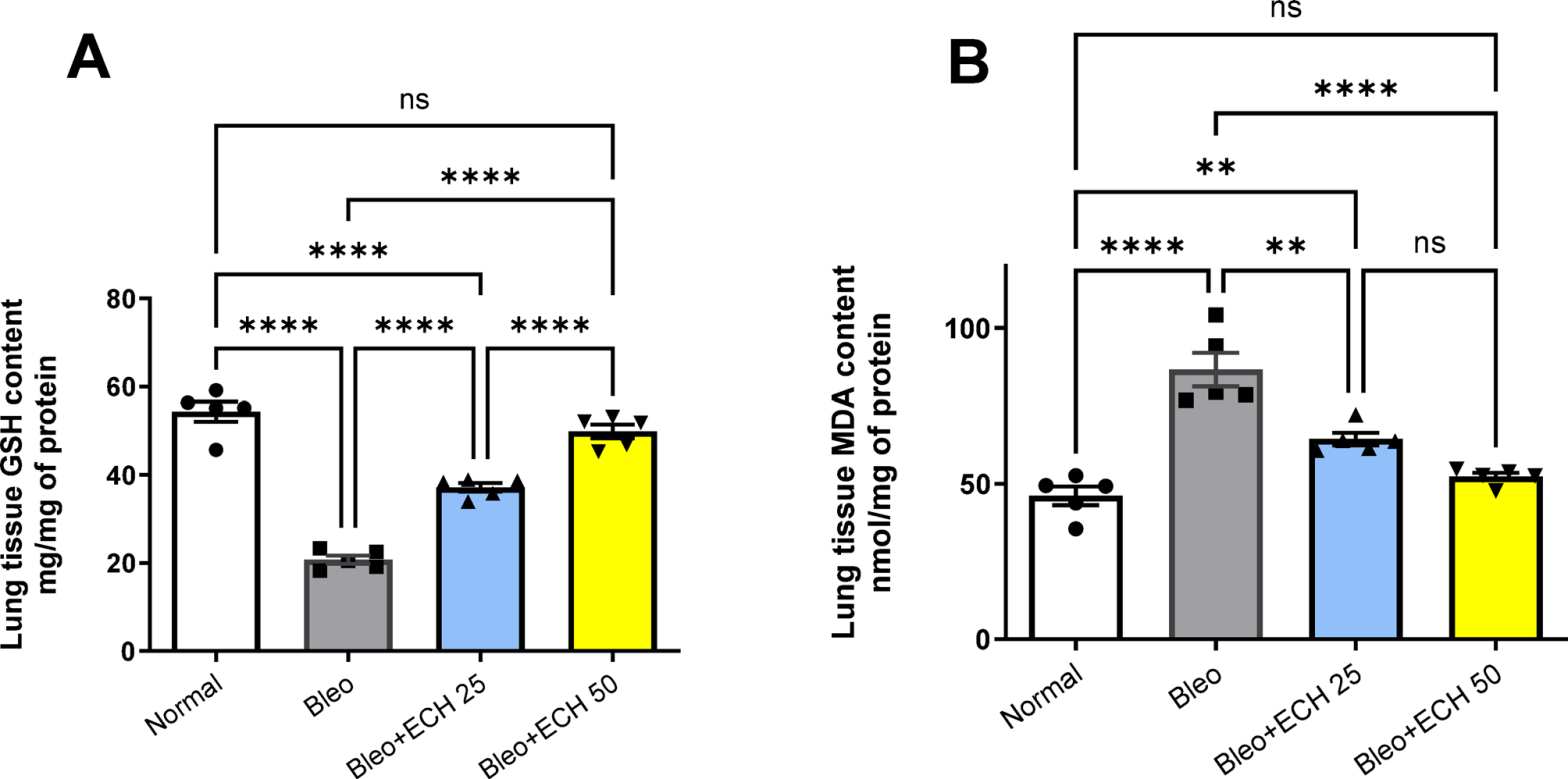
Effects of *Echinacea purpurea* on lung tissue contents of (A) GSH and (B) MDA contents in Bleomycin-induced Pulmonary fibrosis in rats. Data is presented as the mean ± SE. Statistical significance was calculated using one-way ANOVA followed by *Tukey’s* test. (** at *p* ≤ 0.01, **** at *p* ≤ 0.0001).

Intra-tracheal Bleo instillation (5 mg/kg) resulted in a decrease in lung tissue GSH contents (20.77 ± 0.859 mg/mg of protein), accompanied by an increase in lung tissue MDA contents (86.66 ± 4.817 nmol/mg of protein) vs. the normal-control group. Oral ECH treatment (25 &50 mg/kg) caused a significant increase in lung tissue GSH contents to reach 37.17 ± 0.863 & 49.87 ± 1.411; respectively, accompanied by a significant decrease in lung tissue MDA contents to reach 64.43 ± 1.808 &52.38 ± 1.083 vs. the Bleo group, respectively. In addition, ECH treatment (50 mg/kg) restored MDA and GSH contents to their normal levels (Fig. 2).

### *Echinacea ameliorates* the TGF-β, MMP-2, MMP-9 and TIMP-1 in Bleomycin-induced pulmonary fibrosis in rats

Intra-tracheal Bleo instillation (5 mg/kg) elicited a significant rise in lung tissue TGF-β, MMP-2, MMP-9 and TIMP-1 contents to reach 52.77 ± 2.176, 14.56 ± 0.245, 13.72 ± 0.586 & 1.86 ± 0.058 ng/mg of protein, respectively vs. the normal-control group.

**Fig. 3:**
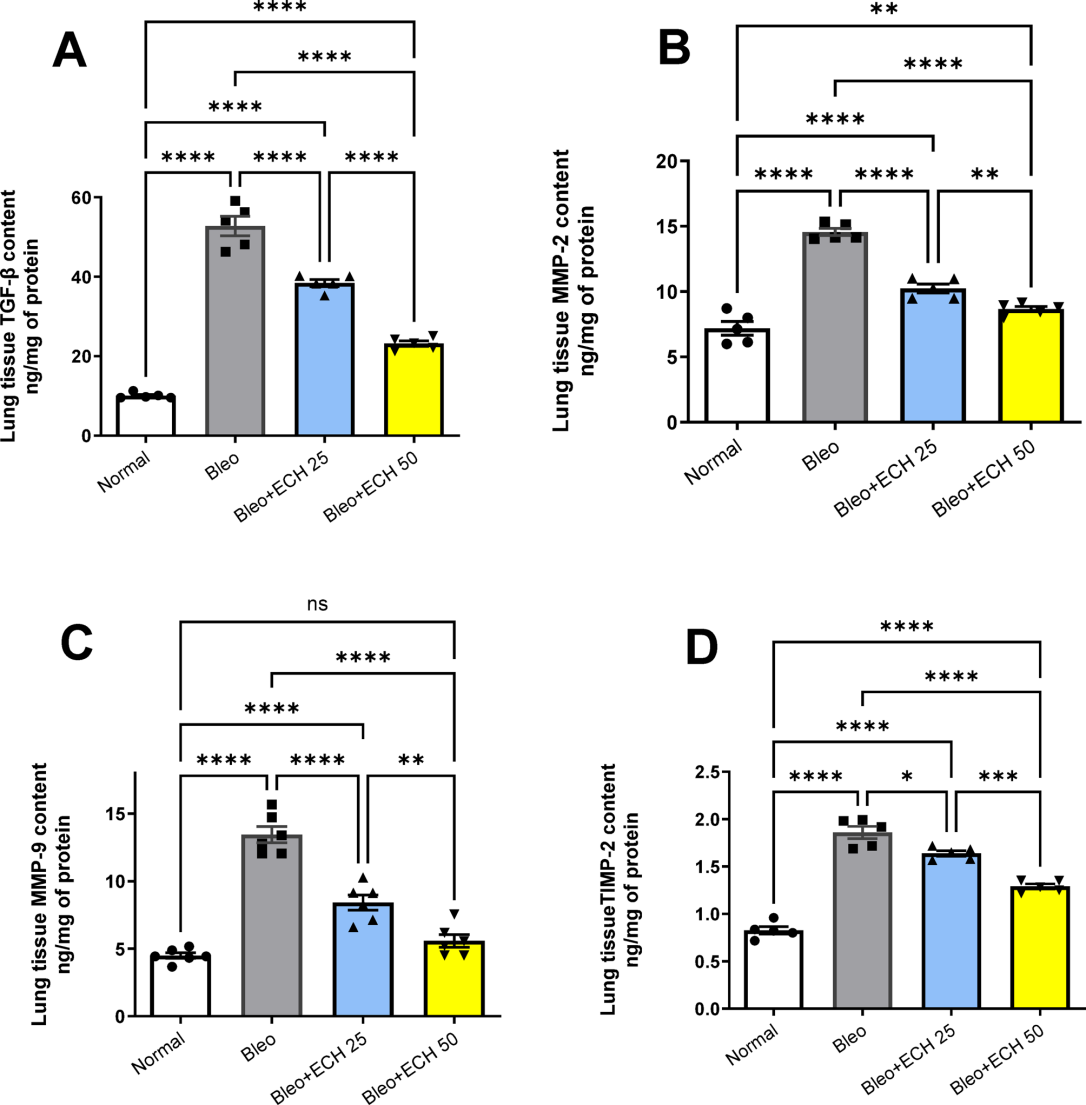
Effects of *Echinacea purpurea* on lung tissue contents of (A) TGF-β, (B) MMP-2, (C) MMP-9 and (D) TIMP-1 contents in Bleomycin-induced Pulmonary fibrosis in rats. Data is presented as the mean ± SE. Statistical significance was calculated using one-way ANOVA followed by *Tukey’s* test. (**** at *p* ≤ 0.0001, *** at *p* ≤ 0.001, ** at *p* ≤ 0.01, * at *p* ≤ 0.05).

Oral ECH treatment (25 mg/kg) elicited a reduction in lung tissue TGF-β, MMP-2, MMP-9 and TIMP-1 contents to reach 38.42 ± 0.807, 10.24 ± 0.302, 8.68 ± 0.547 & 1.64 ± 0.026 respectively vs. the Bleo group. Similarly, Oral ECH treatment (50 mg/kg) elicited a reduction in lung tissue TGF-β, MMP-2, MMP-9 and TIMP-1 contents to reach 23.19 ± 0.622, 8.66 ± 0.181, 5.8 ± 0.458 &1.29 ± 0.024, respectively vs. the Bleo group. Moreover, ECH treatment (50 mg/kg) restored MMP9 content to its normal levels (Fig. 3).

### *Echinacea ameliorates* the MMP-9/TIMP-1 ratio in Bleomycin-induced pulmonary fibrosis in rats

**Fig. 4:**
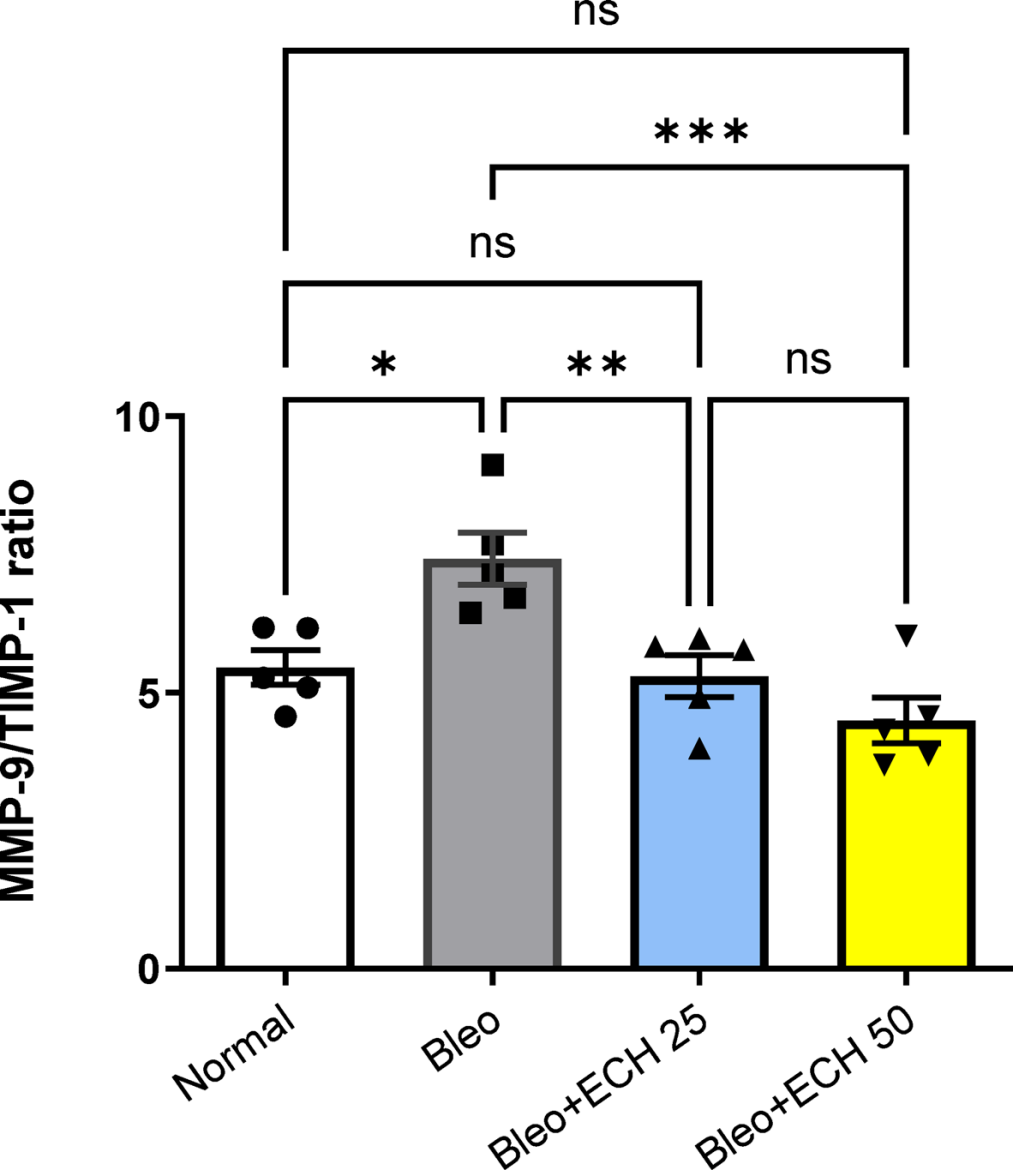
Effects of *Echinacea purpurea* on lung tissue MMP-9/TIMP-1 ratio in Bleomycin-induced Pulmonary fibrosis in rats. Data is presented as the mean ± SE. Statistical significance was calculated using one-way ANOVA followed by *Tukey’s* test. (*** at *p* ≤ 0.001, ** at *p* ≤ 0.01, * at *p* ≤ 0.05).

Intra-tracheal Bleo instillation (5 mg/kg) caused a 142% rise in the MMP-9/TIMP-1 ratio vs. the normal-control group. Oral ECH treatment (25 &50 mg/kg) elicited a 69% and 63% reduction in MMP-9/TIMP-1 ratio vs. the Bleo group. Noticeably, ECH treatment (50 mg/kg) returned MMP9 /TIMP-1 to its normal ratio (Fig. 4).

### *Echinacea ameliorates* collagen-1 and α-SMA in Bleomycin-induced pulmonary fibrosis in rats

**Fig. 5:**
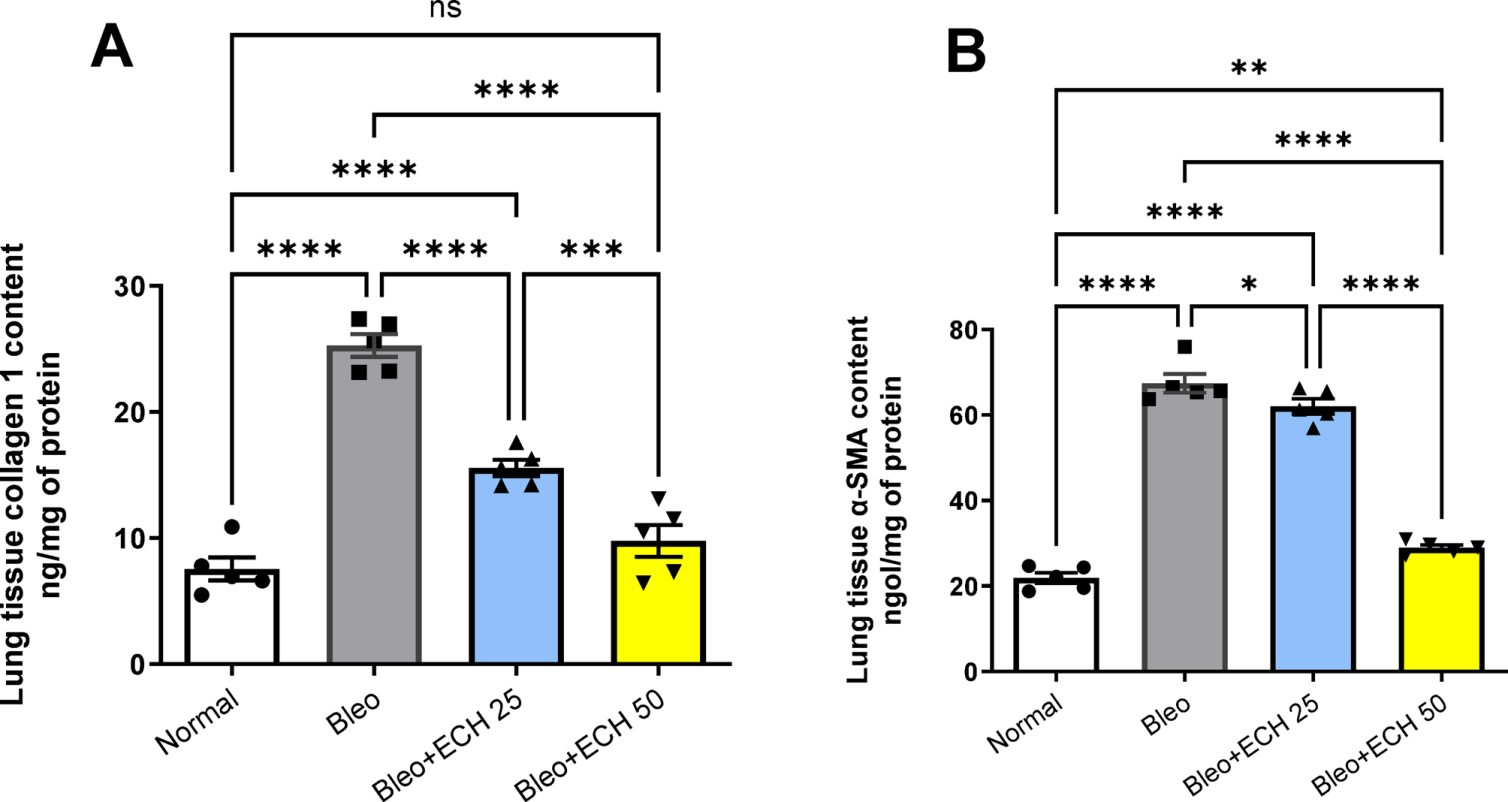
Effects of *Echinacea purpurea* on lung tissue contents of (A) Collagen-1 and (B) α-SMA contents in Bleomycin-induced Pulmonary fibrosis in rats. Data is presented as the mean ± SE. Statistical significance was calculated using one-way ANOVA followed by *Tukey’s* test. (**** at *p* ≤ 0.0001, *** at *p* ≤ 0.001, ** at *p* ≤ 0.01, * at *p* ≤ 0.05).

Intra-tracheal Bleo instillation (5 mg/kg) increased lung tissue collagen-1 and α-SMA contents to reach 25.27 ± 0.805 & 67.44 ± 1.949 ng/mg of protein, respectively vs. the normal-control group. Oral ECH treatment (25 mg/kg) caused a reduction in lung tissue collagen-1 and α-SMA vs. the Bleo group to reach 15.56 ± 0.577 & 62.06 ± 1.56, respectively. Similarly, ECH treatment (50 mg/kg) reduced the lung tissue collagen-1 and α-SMA vs. the Bleo group to reach 9.78 ± 1.127 & 28.99 ± 0.581, respectively. In addition, ECH treatment (50 mg/kg) returned lung tissue collagen-1 to its normal level (Fig. 5).

### Quantitative real-time PCR gene expression of NOX4, CTGF and ET-1 in lung tissues

Lung tissue qRT PCR gene expression of NOX4, CTGF and ET-1 were significantly raised to 2.95 ± 0.05, 3.62 ± 0.033& 3.02 ± 0.052 in the Bleo-control group vs. the normal-control group; respectively.

**Fig. 6:**
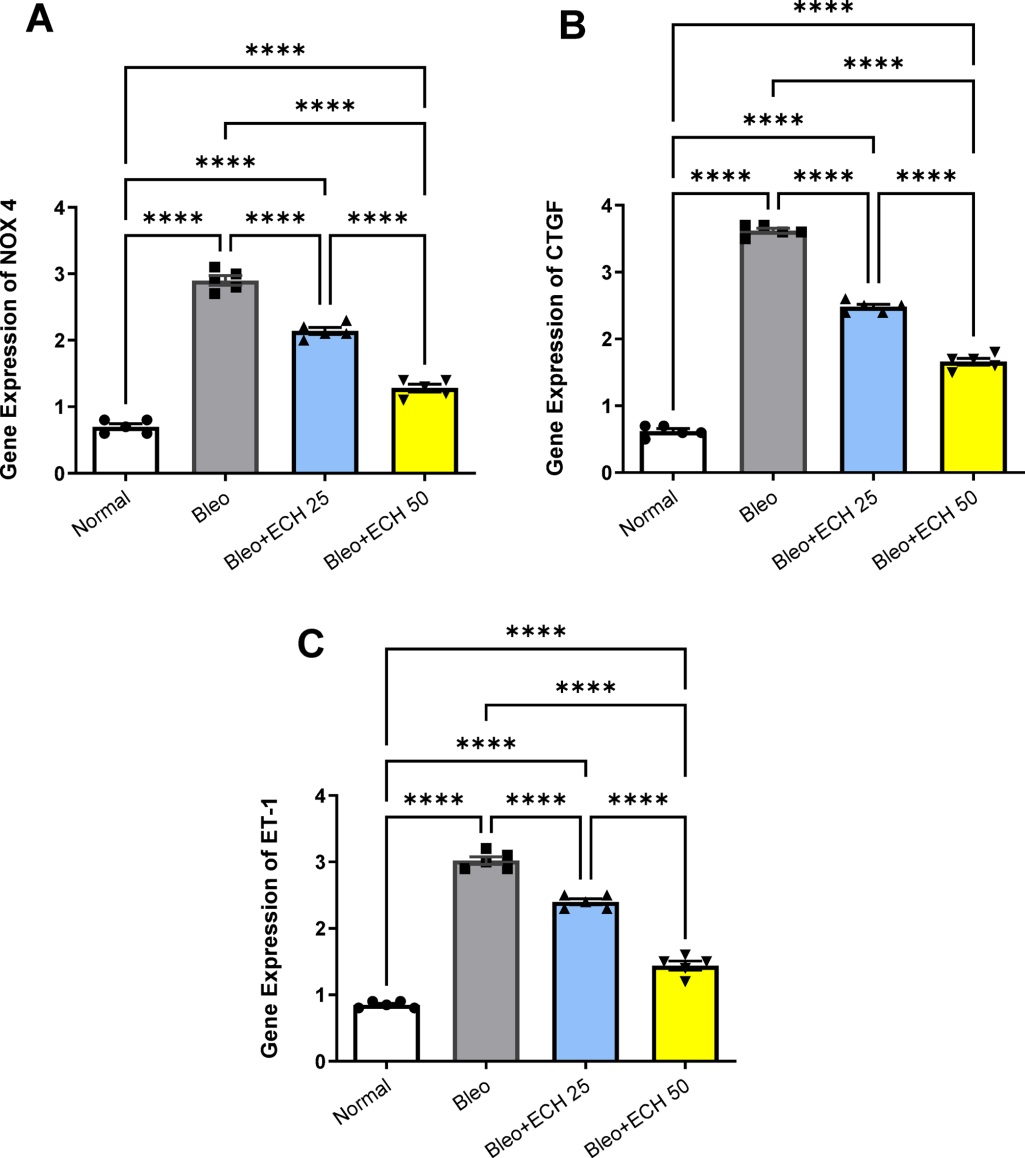
Effects of *Echinacea purpurea* on lung tissue qRT PCR gene expression of (A) NOX4, (B) CTGF and (C) ET-1 in Bleomycin-induced Pulmonary fibrosis in rats. Data is presented as the mean ± SE. Statistical significance was calculated using one-way ANOVA followed by *Tukey’s* test. ( **** at *p* ≤ 0.0001).

Oral ECH treatment (25 mg/kg) decreased the lung tissue gene expression of NOX4, CTGF and ET-1 vs. the Bleo group to reach 2.14 ± 0.046, 2.48 ± 0.033 & 2.4 ± 0.04, respectively. Similarly, ECH treatment (50 mg/kg) decreased the lung tissue gene expression of NOX4, CTGF and ET-1 vs. the Bleo group to reach 1.28 ± 0.052, 1.66 ± 0.046 &1.44 ± 0.061; respectively (Fig. 6).

### Histopathological examination of lung tissues

**Fig. 7:**
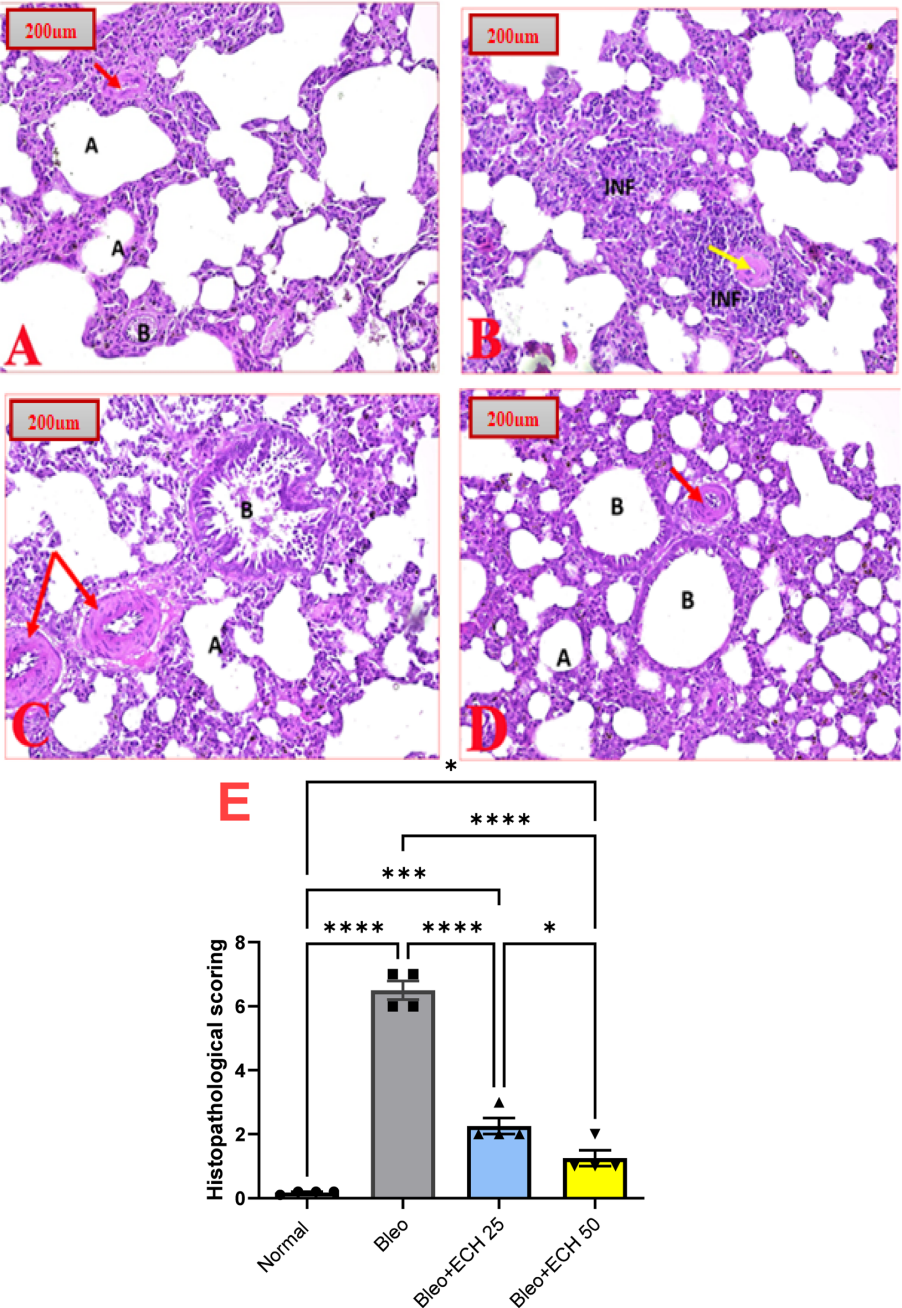
Effects of *Echinacea purpurea* on the histological pictures of lung tissues in rats. **(A)** A photomicrography of lung tissue of the normal-control group showing normal-looking architecture. The alveolar sacs (A) are separated by alveolar septa, the bronchioles (B) have intact walls (Red arrows). **(B)** A photomicrography of lung tissue of the Bleomycin-control group showing damaged bronchioles, inflammatory cells infiltrate (INF) and attenuated alveolar vessels (Yellow arrow). **(C)** A photomicrography of lung tissue of the group treated orally with Echinacea (25 mg/kg) showing alveolar sacs (A) separated by alveolar septa. The bronchioles (B) have intact walls and the bronchial vessels look almost normal (Red arrows). **(D)** A photomicrography of lung tissue of the group treated orally with Echinacea (50 mg/kg) showing alveolar sacs (A) are separated by alveolar septa, bronchioles (B) have intact walls and normal-looking bronchial vessels (Red arrows) (H&E 16x). **(E)** Histopathological scoring of lung tissue of all groups. Data is presented as the mean ± SE. Statistical significance was calculated using one-way ANOVA followed by *Tukey’s* test. (**** at *p* ≤ 0.0001, *** at *p* ≤ 0.001, * at *p* ≤ 0.05).

Figure 7 illustrates the histopathological pictures of the lung tissues of different experimental groups. Pictures of lung tissues of normal-control rats revealed a normal histopathological structure of lungs, bronchia and bronchioles. The alveolar sacs are separated by alveolar septa, comprising alveolar lining cells and thin-walled capillaries. The bronchioles have intact walls with normal-looking ciliated columnar epithelium and normal-looking bronchial vessels (Fig. 7A).

Intra-tracheal Bleo instillation (5 mg/kg) caused substantial lung tissue histopathological alterations as shown by the destruction of the alveolar sacs and bronchioles. the alveolar sacs seem totally destroyed. The bronchioles have damaged walls and inflammatory cells infiltrate, accompanied by attenuated alveolar vessels can be noticed (Fig. 7B).

Oral ECH treatment (25 mg/kg) resulted in a dose-dependent improvement of lung tissues, bronchi and bronchioles. The alveolar sacs are separated by alveolar septa. The bronchioles have intact walls and the bronchial vessels look almost normal (Fig. 7C).

Oral ECH treatment (50 mg/kg) resulted in a normal-looking architecture of the lung.tissues, bronchia and bronchioles. The alveolar sacs are separated by alveolar septa, comprising alveolar lining cells and thin-walled capillaries. The bronchioles have intact walls with normal-looking ciliated columnar epithelium. Also, normal-looking bronchial vessels can be noticed (Fig. 7D).

The severity of alterations in the lung was blindly scored microscopically, and the histopathological scoring of the stained lung tissue is presented in Fig. 7E.

## Discussion

IPF is a deadly chronic fibroproliferative lung disease with idiopathic origins. Bleo-induced pulmonary fibrosis is considered one of the most commonly used in vivo models for the induction of lung fibrosis. Bleo induces cytotoxicity and blocks the synthesis of proteins and DNA, leading to oxidative stress, inflammation and fibrosis of the lung tissues^4^. The current study aims to evaluate the possible protective effects of ECH against Bleomycin-induced pulmonary fibrosis.

In the present study, Intra-tracheal Bleo instillation (5 mg/kg) resulted in significant oxidative stress as manifested by elevated lipid peroxidation products calculated as MDA and reduced the lung tissue contents of GSH. Previous studies linked Bleo administration to the production of oxidative stress parameters such as lipid peroxidation end products (MDA). Moreover, Bleo use decreased the natural antioxidants in the lung tissues (e.g., superoxide dismutase, catalase and GSH), eventually initiating cellular injury^25,26^.

Bleo instillation in the current work caused a significant inflammation as manifested by peaking of lung tissue TGF-β. Cellular injury caused by oxidative stress initiates inflammation via the activation of numerous proinflammatory cytokines and growth factors. Inflammation then leads to the pathophysiological mechanisms of pulmonary fibrosis. TGF-β is a pro-fibrotic growth factor that is considered the major player in pulmonary fibrosis where it leads to substantial inflammation, dysregulated cellular repair and promotes progressive fibrosis, particularly in the early phase^8,27^. TGF-β also suppresses natural autophagy and amplifies the tissue damage response to further promote inflammation and fibrosis via enhancement of fibroblast activation^28^.

Additionally, the current work demonstrated that intra-tracheal Bleo instillation caused an elevation of lung tissue MMP-2, MMP-9 TIMP-1, collagen-1 and α-SMA contents along with MMP-9/TIMP-1 ratio. Bleo also caused a significant increment in PCR expression of NOX4, CTGF as well as ET-1 in lung tissues. Moreover, the histopathological examination of lung tissues of rats treated intratracheally with Bleo showed entirely destructed alveolar sacs, damaged walls of the bronchioles, and attenuated alveolar vessels accompanied by inflammatory cell infiltrate. Previous studies reported that Bleo use causes alveolar inflammation that increases over time. On day 7, fibroblast proliferation was stimulated, inflammation began to worsen, and collagen production surged. On day 14, the alveolar structure was destroyed and collagen began to accumulate widely, displaying the first pathological signs of pulmonary fibrosis^29^. The homeostasis of the lung’s extracellular matrix (ECM) is tightly regulated under normal circumstances. However, in idiopathic pulmonary fibrosis, this homeostasis is seriously dysregulated where ECM accumulation occurs, leading to progressive fibrosis^30^. Lung fibrosis is characterized by an excessive buildup of ECM proteins in the lung interstitium, which is a major cause of lung stiffness, shortness of breath, and ultimately respiratory failure. The ECM sustains mechanical support and functions as a connector between cells, therefore it exerts a crucial role in preserving typical cellular structure and function^31^. The production and deposition of collagen, fibronectin and α-SMA are all increased by abnormal fibroblast activation. The interstitium became rigid as a result of excessive ECM production and deposition^8^. Because of their ability to remove ECM, matrix metalloproteinases have acquired considerable attention in the scope of IPF pathogenesis. MMPs are extracellular enzymes that are initially released as inactive zymogens. MMPs and their tissue inhibitors (TIMPs) are the chief enzymes involved in ECM metabolism^32^. Gelatin, elastin, and other various forms of collagen are particularly susceptible to degradation by MMP-2 and MMP-9, commonly defined as Gelatinases A and B; respectively. Both proteases are found in the lungs of idiopathic pulmonary fibrosis patients and are expressed by bronchial, bronchiolar fibroblasts, fibrocytes, and alveolar epithelial cells. The fact that the deletion of MMP-2 may be functionally compensated for by MMP-9 assesses a direct contribution of one single gelatinase to lung fibrogenesis rather challenging^33^. TIMP-1 is a specific tissue inhibitor of MMP-9 while TIMP-2 is a specific tissue inhibitor of MMP-2^34^. A balanced MMP/TIMP ratio in the lung tissue is essential for balancing the synthesis and breakdown of the ECM^31^. Numerous studies reported the elevation of gelatinases and their tissue inhibitors in lung fibrosis induced experimentally via lipopolysaccharide^34^ and paraquat^35^. Also, many studies demonstrated elevation of MMP-2, MMP-9 and TIMP-1 in Bleo-induced lung fibrosis^27,29,32,36^. Similar to the current work, Bai et al.. (2020) reported that Bleo instillation was linked to elevated expressions of fibrosis-related biomarkers, viz., α-SMA and collagens I, II &III. The key ECM elements of the lungs are collagen I and collagen III, which are important fibroblast protein signals. Collagens are markedly increased in lung fibrosis, with collagen I being the most markedly accentuated member of the group^37^.

Seven NOX homologs make up the NADPH oxidases (NOXs) family. NOX4 has been linked to many fibrotic disorders, including hepatic, renal, cardiac, dermal and pulmonary fibrosis. It is well documented that NOX4, along with the pro-fibrotic and pro-proliferative endothelin-1 (ET-1) and connective tissue growth factor (CTGF), are all considerably upregulated in pulmonary fibrosis^38^. NOX4 is a key player in the pathophysiology of IPF. Moreover, it is excessively expressed in the lungs post-Bleo administration in experimental animals and in humans of BLM-subjugated. TGF-β is the major contributor to NOX4 expression. α-SMA and collagen expression are modulated by NOX4 in lung fibroblasts^39^. Additionally, TGF-β induces the expression of two crucial matricellular proteins, CTGF and ET-1, which work downstream of this cytokine to stimulate fibrogenic responses such as myofibroblast development, collagen synthesis, and α-SMA expression. There is accumulating evidence that CTGF and ET-1 are increased during fibrogenesis. The CTGF and ET-1 may be possible targets for anti-fibrotic therapy, according to a growing body of research showing that TGF-β, CTGF, and ET-1 expressions are cooperatively increased within the lung tissue in IPF^40^. Many studies report a rise in CTGF and ET-1 expression levels in pulmonary tissues in response to Bleo administration^40–42^.

Recent studies demonstrate that in pulmonary fibrosis, ET-1, CTGF, and the balance between MMPs and their tissue inhibitors TIMPs interact in a complex crosstalk that drives the progression of the disease^13^. ET-1, a potent vasoconstrictor and pro-fibrotic factor, promotes the activation of fibroblasts and their differentiation into myofibroblasts, which are key contributors to excessive ECM deposition. ET-1 also stimulates the expression of CTGF, a critical mediator of fibrosis that enhances fibroblast proliferation and collagen synthesis^43^. Meanwhile, the MMPs, particularly MMP-2 and MMP-9, are responsible for ECM degradation, but their activity is tightly regulated by TIMPs. In fibrotic lungs, the balance between MMPs and TIMPs is often skewed towards increased TIMP levels, resulting in reduced ECM turnover and excessive collagen accumulation. The interplay between ET-1 and CTGF exacerbates this imbalance by further promoting ECM production and inhibiting its degradation, thus amplifying the fibrotic response and contributing to the progressive nature of pulmonary fibrosis^44^.

Since medicinal plants have a variety of chemical components and exhibit a variety of biological functions, they are frequently employed in medicine as alternative therapies and/or supplementary therapeutic regimens. *Echinacea purpurea* (ECH) is a herbaceous perennial flowering plant that has antiviral, anti-fibrotic as well as antioxidant properties^15^.

In the present work, ECH treatment (25 and 50 mg/kg; p.o.) resulted in a significant reduction in the lung tissue contents of MDA, TGF-β, MMP-2, MMP-9, TIMP-1, MMP-9/TIMP-1 ratio, collagen-1 and α-SMA. ECH treatment also caused a significant increment in the lung tissue GSH contents. Moreover, ECH significantly reduced the PCR expression of NOX4, CTGF as well as ET-1 in lung tissues and mitigated the lung tissue histopathological fibrotic changes induced by Bleo administration. Taken together, these results suggest that ECH ameliorates oxidative Stress, inflammation, histopathological changes and lung fibrosis in Bleo-induced pulmonary fibrosis in rats.

Few studies explored the anti-fibrotic effects of ECH on lung fibrosis. In one study, ECH is reported to have anti-inflammatory properties and thus mitigates lipopolysaccharide-induced lung damage via the reduction of inflammation, apoptosis, and the activation of the TLR4/NF-κB signaling pathway^45^. Guo et al.. (2023) reported that ECH suppresses NO Production in lipopolysaccharide-induced lung injury via the inhibition of NLRP3^46^. Moreover, ECH is reported to minimize IL-6 and TNF-α concentrations in patients suffering from viral and bacterial respiratory infections. ECH boosts antioxidant capacity in many tissues including the brain liver, heart, kidneys and lungs. ECH also inhibits the proinflammatory cytokines, viz., Nrf2, HO-1 and NF-κB. ROS production, inflammation, cell proliferation and NADPH oxidase expression are also modulated by ECH^16^. Another recent study reported a significant improvement in lung histopathological changes post-treatment with ECH in diabetic male rats as manifested by the substantial reduction in the interalveolar septal thickness, collagen fibers percentage as well as caspase-3 expression in lung tissue^47^.

The current study, while demonstrating promising anti-fibrotic effects of ECH in Bleo-induced pulmonary fibrosis rat model, is subject to several limitations that may affect the generalizability and translational potential of its findings. Species-specific differences between rats and humans limit the direct applicability of results to human clinical settings. The absence of the effect of environmental and confounding factors like concurrent diseases also limits the generalizability of the study. The study also lacks a comprehensive dose-response assessment, evaluating only two ECH doses (25 and 50 mg/kg). Importantly, no human data are available to support clinical translation, and the short-term design fails to assess long-term efficacy or safety. Addressing these limitations in future research will be crucial to validating ECH as a viable therapeutic agent for pulmonary fibrosis.

To the best of the author’s knowledge, this is the first study into the potential therapeutic benefits of ECH on Bleo-induced lung fibrosis. Furthermore, the potential molecular mechanisms underlying these beneficial actions have been explored.

## Conclusion

Taken together, we have revealed the potential anti-oxidant, anti-inflammatory and anti-fibrotic effects of *Echinacea purpurea* (ECH) against Bleomycin-induced pulmonary fibrosis in Rats. ECH treatment resulted in a significant reduction in the lung tissue contents of MDA, TGF-β, gelatinases (MMP-2& MMP-9) along with their inhibitor (TIMP-1), MMP-9/TIMP-1 ratio, collagen-1 and α-SMA. ECH treatment also caused a significant increment in the lung tissue GSH contents. Moreover, ECH significantly reduced the PCR expression of NOX4, CTGF as well as ET-1 in lung tissues and mitigated the lung tissue histopathological fibrotic changes induced by Bleo administration. Accordingly, ECH is anticipated as a potential therapy due to its anti-oxidant, anti-inflammatory and anti-fibrotic effects and thus it can be added to the treatment regimen of pulmonary fibrosis. Further investigations are necessary to consider adding ECH to the treatment protocol for pulmonary fibrosis.

## Data Availability

All Data will be available upon request. Please contact the corresponding author R.E. Mostafa if someone wants to request the data from this study.

## Abbreviations

IPF: Idiopathic pulmonary fibrosis
Bleo: Bleomycin
ECH: Echinacea purpurea
COPD: Chronic obstructive pulmonary disease
TGF-β1: Tissue growth factor-beta1
ROS: Reactive oxygen species
MMP-2: Matrix metalloproteinases 2
MMP-9: Matrix metalloproteinases 9
TIMP-1: Tissue Inhibitor of metalloproteinase 1
α-SMA: Alpha-Smooth muscle actin
MDA: Malondialdehyde
GSH: Reduced glutathione
NOX4: NADPH oxidase 4
CTGF: Connective tissue growth factor
ET-1: Endothelin-1
